# Mapping of the gene in tomato conferring resistance to root-knot nematodes at high soil temperature

**DOI:** 10.3389/fpls.2023.1267399

**Published:** 2023-10-10

**Authors:** Zübeyir Devran, Tevfik Özalp, David J. Studholme, Mahmut Tör

**Affiliations:** ^1^Department of Plant Protection, Faculty of Agriculture Akdeniz University, Antalya, Türkiye; ^2^Department of Entomology, Directorate of Plant Protection Research Institute, Bornova, İzmir, Türkiye; ^3^Biosciences, University of Exeter, Exeter, United Kingdom; ^4^Department of Biological Sciences, School of Science and the Environment, University of Worcester, Worcester, United Kingdom

**Keywords:** high soil temperature, gene mapping, *Meloidogyne incognita*, *RRKN1*, SNP

## Abstract

Root-knot nematodes (RKNs, *Meloidogyne* spp.) can cause severe yield losses in tomatoes. The *Mi-1.2* gene in tomato confers resistance to the *Meloidogyne* species *M. incognita*, *M. arenaria* and *M. javanica*, which are prevalent in tomato growing areas. However, this resistance breaks down at high soil temperatures (>28°C). Therefore, it is imperative that new resistance sources are identified and incorporated into commercial breeding programmes. We identified a tomato line, MT12, that does not have *Mi-1.2* but provides resistance to *M. incognita* at 32°C soil temperature. An F_2_ mapping population was generated by crossing the resistant line with a susceptible line, MT17; the segregation ratio showed that the resistance is conferred by a single dominant gene, designated *RRKN1* (*Resistance to Root-Knot Nematode 1*). The *RRKN1* gene was mapped using 111 Kompetitive Allele Specific PCR (KASP) markers and characterized. Linkage analysis showed that *RRKN1* is located on chromosome 6 and flanking markers placed the locus within a 270 kb interval. These newly developed markers can help pyramiding *R*-genes and generating new tomato varieties resistant to RKNs at high soil temperatures.

## Introduction

Tomato (*Solanum lycopersicum*) is an economically important vegetable crop grown in regions with warm and temperate climates. Root-knot nematodes (RKNs) are one of the most important pests of tomato plants and are obligate endoparasites that can cause galls on the roots of the plant they feed on and restrict the uptake of water and nutrients from the vascular bundles. Therefore, they cause a decrease in yield and quality (Williamson and Hussey, 1996).

Management methods for RKNs include crop rotation with resistant varieties or less- susceptible crops (Talavera et al., 2009; Abd-Elgawad, 2022), cultural and tillage practices (Marquez and Hajihassani, 2023), microbial biocontrol agents (Hashem and Abo-Elyousr, 2011) and nematicides (Rawal, 2020; Phani et al., 2021). Using resistant varieties is one of the most common and successful control methods today due to their lack of residue, ease of application and environmental friendliness (Devran and Söğüt, 2010). Plant disease resistance genes have been classified into eight major families based on their amino acid motif organization and their membrane spanning domains (Gururani et al., 2012). The majority of resistance genes encode proteins belonging to the toll/interleukin1 receptor (TIR) – nucleotide binding site – leucine-rich repeat (TIR-NBS-LRR) or intracellular coiled coil (CC) –nucleotide binding site – leucine-rich repeat (CC-NBS-LRR, Mchale et al., 2006), which are now called NLRs (Barragan and Weigel, 2021). The *Mi-1.2* gene encodes a CC-NLR protein (Milligan et al., 1998) and provides effective protection against *Meloidogyne incognita*, *M. javanica* and *M. arenaria*, which are commonly present in tomato growing areas (Roberts and Thomason, 1986). However, the *Mi-1.2* gene loses its effect at high soil temperatures and the resistance breaks down (Dropkin, 1969; Özalp and Devran, 2018). Especially in hot climates, the increase in soil temperatures in summer plantings creates problems for growers using tomato varieties carrying the *Mi-1.2* gene. This climate-derived condition severely limits the use of resistant tomato cultivars carrying the *Mi-1.2* gene. Therefore, it is imperative to identify new resistance gene(s) for the sustainability of resistance against RKN. Gene(s) conferring resistance at high soil temperature may allow earlier planting and prolong the growing period in hot climates regions. Therefore, researchers have identified new resistance genes from *Mi-2* to *Mi-9*. Among these genes, *Mi-2*, *Mi-3*, *Mi-4*, *Mi-5*, *Mi-6* and *Mi-9* were found to be effective at high soil temperatures (Cap et al., 1993; Yaghoobi et al., 1995; Veremis and Roberts, 1996a; Veremis and Roberts, 1996b; Veremis et al., 1999; Ammiraju et al., 2003). Of these genes, *Mi-2* and *Mi-4* have not yet been mapped; *Mi-3* and *Mi-5* have been mapped to chromosome 12 (Yaghoobi et al., 1995; Ammiraju et al., 2003; Jablonska et al., 2007), while *Mi-1*, *Mi-6* and *Mi-9* have been mapped to chromosome 6 (Veremis and Roberts, 1996b; Kaloshian et al., 1998; Milligan et al., 1998; Ammiraju et al., 2003; Jablonska et al., 2007). Gene *Mi-3* additionally provides resistance against *Mi-1.2*-virulent RKN populations (Yaghoobi et al., 1995), while *Mi-6* and *Mi-9* are susceptible to *Mi-1.2*-virulent RKN populations (Veremis and Roberts, 1996b; Veremis and Roberts, 2000). Apart from these genes, *Mi-HT* provides durable resistance at high temperatures and shows homology to *Mi-1.2* and *Mi-9* (Wu et al., 2009). However, it is not known whether *Mi-HT* is a different gene from *Mi-9* (Wang et al., 2013). The *Mi-9* gene was recently characterized and cloned by Jiang et al. (2023) using a chromosome-scale genome assembly of *Solanum arcanum* LA2157. Seven candidate genes were determined using comparative genomic studies and markers shown to be linked to the *Mi-9* gene. Transcriptomics demonstrated that five of the seven candidate genes were expressed in root tissues. Cloning of the *Mi-9* gene was confirmed by silencing candidate genes and investigating the resistance performance of plants obtained. In addition, *Mi-9* was transformed into susceptible tomato lines and was demonstrated to confirm resistance to RKN at high soil temperature (Jiang et al., 2023). Previously, we investigated the response of the *Mi-1.2* gene at high soil temperatures and observed that the resistance provided by the *Mi-1.2* gene broke down in plants exposed to 32°C soil temperature for 48 hours or longer (Özalp and Devran, 2018). We then proceeded to investigate the response of the identified tomato line along with the other tomato lines at high soil temperature. In this study, we determined that the tomato line MT12 confers resistance to the RKN, and the durability provided was heat stable. Thus, we focused our further studies on this line. We used next generation sequencing (NGS) technology to map this new gene in MT12 designated *RRKN1* (*Resistance to Root-Knot Nematode 1*), and identified the genomic interval on the chromosome 6 for the *RRKN1* using Kompetitive Allele Specific PCR (KASP) markers.

## Materials and methods

### Plant materials

The parental genotypes MT12 and MT17 were obtained from tomato genetic resources of Multi Tohum (Antalya, Turkey). The tomato line MT12 was used as a resistance source in this study. There is not much information about the genetic background of this genotype. MT17 is a susceptible parent obtained from female (*Solanum lycopersicum* X *S. hirsutum*) and male (*Solanum lycopersicum* X *Solanum pimpinellifolium*). Tomato cultivars Seval F_1_ carrying *Mi-1.2* gene and susceptible Tueza F_1_ (Multi Tohum, Antalya, Turkey) were used as controls in the experiments. The presence or absence of *Mi-1.2* gene in all tomato plants was confirmed using Mi23 primer set (Seah et al., 2007) (**Supplementary Figure 1**).

### Nematode isolates

Two avirulent isolates of *M. incognita*, S6 and K7, were used in the experiment to investigate the response of the MT12 at a high temperature (Devran and Sogut, 2009). All isolates used have been continued as pure nematode cultures since 2008 in Devran laboratory. The responses of MT12 to *Mi-1.2* natural virulent populations V12 and V21 of *M. incognita* were also investigated. Only the *M. incognita* avirulent S6 isolate was used in the mapping and inheritance studies of *RRKN1.*

Avirulent isolates were multiplied on susceptible tomato cultivar Tueza F_1_, while *Mi-1.2* natural virulent isolates were multiplied on resistant tomato cultivar Seval F_1_ containing the *Mi-1.2* gene to maintain their virulence in a growth chamber at 25°C ± 0.5 with a 16:8 hours photoperiod (light: dark) and 65 ± 5% relative humidity. Sixty days after inoculation the plants were removed, and the plant roots were washed with water. Egg masses were collected from the root using a small needle and placed in centrifuge tubes (Özalp and Devran, 2018). They were then stored at 4°C until inoculation.

### Testing plant lines with nematode

Tomato seeds were sown in vials including vermiculite, perlite, and peat (v:v:v: 1:1:1) and maintained in a seedling facility. Seedlings with four true leaves were transplanted into 250 ml plastic pots including sterilized sandy soil and were kept in the growth chamber at 25°C for a week to ensure root development. For the tests to be carried out at high temperature, the pots were transferred to a growth chamber at 32°C. Soil temperature was monitored with the probes placed in pots and nematodes were inoculated when the soil temperature reached 32°C. Seedlings were exposed to soil temperatures at 32°C for 7 days.

For nematode inoculation, egg masses were placed in a sieve and fresh second stage juveniles (J2s) from hatched eggs were collected and counted under a light microscope and diluted to 1000 J2s/ml. A total of 1000 J2s were inoculated into two holes (0.5 ml of water per hole) near the stem of each plant. The procedure was performed according to former studies (Öçal et al., 2018; Nas et al., 2023). After seven days, the plants were transferred to a growth chamber at 25°C ± 0.5 with a 16:8 hours photoperiod (light: dark) and 65 ± 5% relative humidity until the end of the experiment.

### Investigating MT12 response to avirulent and *Mi-1.2* natural virulent isolates at high soil temperature

To observe the response of the MT12 tomato line to nematode isolates at a high temperature, an experiment was conducted under 25°C and 32°C soil temperature conditions. For this purpose, avirulent *M. incognita* isolates K7 and S6 were used for the inoculation of plants. Besides resistant source MT12, susceptible tomato cultivar Tueza F_1_ and resistant tomato cultivar Seval F_1_ were used as control plants in the experiment.

To observe the response of the MT12 tomato cultivar to *Mi-1.2* natural virulent *M. incognita* isolates, an experiment was conducted under 25°C soil temperature conditions. For this purpose, *Mi-1.2* natural virulent *M. incognita* isolates V12 and V21 were used for the inoculation of plants.

Both experiments were performed as described above for testing plant lines. The experiments were carried out as five replicates and two repeats according to the completely randomized design.

### Inheritance and mapping population

A cross between the resistant and susceptible parent lines MT12 and MT17, respectively, was generated and the F_1_ line was obtained. An F_2_ mapping population was generated by selfing the F_1_ line. A total of 130 F_2_ seeds were obtained from a single F_1_ tomato plant and were used in the phenotyping and genotyping experiments.

### Data evaluation and statistics

Plants were uprooted sixty days post inoculation (dpi) and the roots were washed under the tap water. The roots were then stained with Ploxine B, and the galls and egg masses were counted and recorded (Öçal et al., 2018). Egg masses and galls in the roots of the plants were counted under a light microscope.

Plants in mapping population were classified as resistant if the individual root system had less than 25 egg masses, or susceptible if the individual root system had 25 or more egg masses (Veremis et al., 1999; Ammiraju et al., 2003; Wang et al., 2013).

Data were analyzed by analysis of variance (ANOVA). Differences among means were examined using the Tukey multiple comparison test. Chi-square tests for specific proportions for goodness of fit were carried out using the PROC FREQ function. All statistical analysis was performed using with the SAS statistical program (v. 9.0 for Windows; SAS Institute Inc., Cary, NC, USA).

### DNA isolation

Genomic DNA was extracted from young leaves collected from parental lines and F_2_ plants using the Wizard Magnetic Kit (Promega) following the manufacturer’s instructions. DNA was then run on an agarose gel and checked for degradation or their high molecular weight. The quality control (QC) of the DNA was determined by Novogene UK (Cambridge, United Kingdom) before proceeding to the sequencing.

### Genome sequencing

The sequencing has been carried out by Novogene UK (Cambridge, United Kingdom), generating 150 bp paired-end read data for each parent line (resistant and susceptible) with NovaSeq 6000.

### Bioinformatics and NGS analysis

As previously described (Kahveci et al., 2021), we took the NGS analysis approach and trimmed the raw reads using BBDuk (filterk=27, trimk=27; https://sourceforge.net/projects/bbmap/) to remove Illumina adapters and to quality-trim both ends to Q12. Trimmed sequences from parental lines were then mapped onto chromosomes 1 – 12 of the SL3.1 version of the available reference tomato genome (*S. lycopersicum* cultivar Heinz 1706, GenBank: GCA_000188115.4) using BBMap (https://sourceforge.net/projects/bbmap/) and the alignment data were converted to the BAM format (Li et al., 2009). The variant detection was performed using BCFtools (Li et al., 2009) and SNPsFromPileups (https://github.com/davidjstudholme/SNPsFromPileups) as previously described (Devran et al., 2018; Kahveci et al., 2021). Integrative Genomics Viewer (IGV) was used to visualize the alignment results (Robinson et al., 2011). We used the Solanaceae Genomics Network web portal (Fernandez-Pozo et al., 2015) to browse and visualize the tomato genomic interval and identify the genes contained therein.

### Strategies for mapping the *RRKN1* gene

At the beginning of the study, there was no information on the location of the *RRKN1* gene. Therefore, we used three different strategies to determine the *RRKN1* locus.

#### Strategy 1: focusing on chromosome 6

Previous studies reported that some genes providing resistance to RKNs in tomatoes are located on chromosome 6 (Milligan et al., 1998; Jablonska et al., 2007; Du et al., 2020). Therefore, initially, we focused on this chromosome. We selected 55 SNPs from the SolCap Tomato Genotyping Panel (https://www.biosearchtech.com) on chromosome 6, nine of which were polymorphic between parental lines (**Supplementary Table 1**).

#### Strategy 2: global mapping

Analysis of the genomic sequences of our parental lines identified 34 new SNPs, distributed over 10 chromosomes (**Supplementary Table 2**). Single-nucleotide differences among parents were visually inspected using IGV. In determining the number of primers to represent each chromosome, we considered the length of each chromosome in the reference genome of the tomato.

#### Strategy 3: information-led mapping

Since the data obtained from the first and second strategies provided information that the gene is most likely to be located on chromosome 6, we focused on this chromosome. Thus, genome sequences belonging to the parents were compared with the reference genome in the IGV and 17 new further SNPs were taken towards marker development and fine mapping (**Supplementary Table 2**).

For all three strategies, initially identified SNPs were used to detect polymorphisms between parental lines and then polymorphic markers were used to screen mapping population.

### Development of KASP markers for *RRKN1* gene

Genomic sequences flanking and including SNPs were determined using Geneious Prime 2019 (Biomatters) and sent to LGC Biosearch Technologies (https://www.biosearchtech.com) to develop KASP primers. The KASP reactions were performed according to previous studies (Devran and Kahveci, 2019; Kahveci et al., 2021) and carried out using the Hydrocycler (LGC Biosearch Technologies, UK) with a 61-55°C touchdown protocol. The fluorescent readings were performed for 2 min at 25°C using a FluOstar Omega Microplate Reader (BMG LABTECH Ortenberg, Germany).

### Accession numbers

The raw sequence reads of parent lines have been deposited in the Sequence Read Archive (SRA) under accession numbers SRR25056976 and SRR25056975 and are accessible via BioProject accession PRJNA988534.

## Results

### Tomato line MT12 does not carry *Mi-1.2* but provides resistance to *Mi-1.2*-avirulent isolates at high soil temperature

In this experiment, we investigated changes in the resistance status of tomato plants inoculated with *M. incognita* K7 and S6 isolates when exposed to soil temperatures of 25°C and 32°C. MT12 (*mi-1.2/mi1.2*) and Seval (*Mi-1.2/mi-1.2*) F_1_ plants were resistant to both isolates at 25°C soil temperature, whilst the Tueza (*mi1.2/mi-1.2*) F_1_ cultivar was susceptible, exhibiting a large number of galls and egg masses, as expected (see **Supplementary Figure 1** for genotypes of plant lines used). No significant differences were observed in the gall and egg mass numbers of both isolates on MT12 and Seval F_1_ cultivar when exposed to 25°C soil temperature (p ≤ 0.05) (**Table 1**). Among the plants exposed to 32°C soil temperature, the highest egg mass and gall number was counted in Tueza F_1_. Resistance was broken at 32°C in Seval F_1_ carrying the *Mi-1.2* gene; however, the gall and egg mass numbers were about half of those observed in the susceptible Tueza F1. Conversely, MT12 showed a resistant response against K7 and S6 isolates despite being exposed to 32°C soil temperature for one week (**Table 1**).

**Table 1:** Number of egg masses and galls of *Meloidogyne incognita* isolates on tomato cultivars exposed to 32°C and 25°C.

Tomato Lines	*M. incognita* K7 isolate				*M. incognita* S6 isolate			
	Egg mass		Gall		Egg mass		Gall	
	32°C	25°C	32°C	25°C	32°C	25°C	32°C	25°C
MT12	6.7 c	1.5 b	5.5 c	1.8 b	7.5 c	1.3 b	7.5 c	3.1 b
Seval F1	57 b	1.1 b	68.4 b	0.6 b	68 b	2.5 b	89.4 b	3.5 b
Tueza F1	127.4 a	419 a	114.9 a	281.3 a	113.2 a	156.7 a	181.5 a	195.7 a

Means within a column followed by the same letter are not significantly different (p ≤ 0.05) according to Tukey’s multiple comparison test.

MT12: Resistant parent.

Seval F1: Tomato cultivar carrying *Mi-1.2* gene.

Tueza F1: Susceptible tomato cultivar.

### Tomato line MT12 is susceptible to naturally *Mi-1.2*-virulent isolates

We also tested MT12 with nematode isolates that are naturally *Mi-1.2*-virulent at 25˚C soil temperature. The *Mi-1.2* naturally virulent isolates V12 and V21 multiplied very well and produced egg masses on the roots of MT12, indicating that MT12 did not provide resistance to *Mi-1.2* naturally virulent isolates. Also, Seval F_1_ lines bearing the *Mi-1.2* gene were overcome by the *Mi-1.2*-virulent isolates as expected. The isolates produced egg masses on roots of susceptible Tueza F_1_ and caused galls. No statistically significant differences were observed in the numbers of galls between the two virulent isolates in the three tested plant lines (p ≤ 0.05) (**Table 2**).

**Table 2:** Number of egg masses and galls of *Mi-1.2*-natural virulent *Meloidogyne incognita* isolates on tomato cultivars.

Tomato Line	*M. incognita* V12 isolate		*M. incognita* V21 isolate	
	Egg mass	Gall	Egg mass	Gall
MT12	356.7 a	200.9 a	203.5 b	166.8 a
Seval F1	283 b	206.5 a	202.3 b	152.1 a
Tueza F1	264.1 b	197 a	301.6 a	177.8 a

Means within a column followed by the same letter are not significantly different (p ≤ 0.05) according to Tukey’s multiple comparison test.

MT12: Resistant parent.

Seval F1: Tomato cultivar carrying *Mi-1.2* gene.

Tueza F1: Susceptible tomato cultivar.

### Resistance to *M. incognita* is controlled by a single dominant gene

We tested the parents and F_1_ plants derived from the cross between the susceptible and resistant tomato lines for resistance to *M. incognita* at 32°C soil temperature. The F_1_ individuals were resistant to *M. incognita*. The F_1_ plant was selfed to obtain F_2_ population. Of 130 F_2_ individuals subjected to *M. incognita*, screening showed 91 resistant and 39 susceptible plants, giving a segregation ratio of 3:1 resistant:susceptible (χ2 = 1,73, p=0.18). This indicates that the resistance is controlled by a single dominant gene designated ***R*** *esistant to* ***R*** *oot-****K*** *not* ***N*** *ematode* ***1*** (*RRKN1)* at 32°C soil temperature.

### Mapping *RRKN1* gene

Initially 55 SNPs on chromosome 6 of the tomato genome from SolCap were selected and converted to KASP markers. We first screened parents with these markers for polymorphism and 9 of them were found to be polymorphic (**Supplementary Table 1**). Then, F_2_ populations were screened with these polymorphic markers. Phenotype and genotype data indicated that *RRKN1* may be located on chromosome 6.

**Figure 1:**
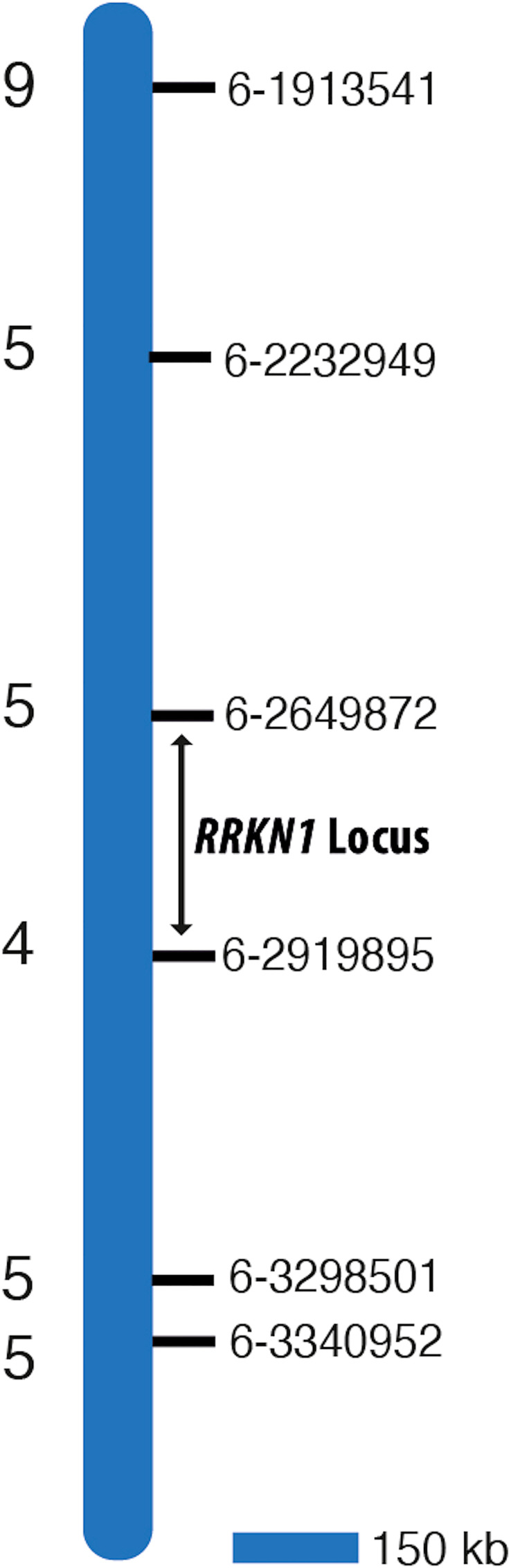
Map interval of *RRKN1* on tomato chromosome 6. Coordinates of molecular markers used to map the *RRKN1* locus on the reference tomato genome SL3 are given on the right of the bar. Numbers on the left of the bar indicate the number of recombinants in 130 F_2_ individuals.

We also took a global genome mapping approach. We developed KASP markers for all chromosomes, except for 8 and 9, for which no SNPs were identified. Markers (**Supplementary Tables 2**, **3**) were first used to screen parents to confirm polymorphism and the F_2_ mapping population was screened. Results showed that only those KASP markers developed from chromosome 6 were linked to the pathological data for *RRKN1.* These findings supported our initial results where we used 9 KASP markers developed from SolCap. Therefore, we focused on chromosome 6 to confirm the location of *RRKN1* gene and developed further new 17 KASP markers from chromosome 6 (**Supplementary Table 2**) to fine map the gene. Two flanking markers, KASP-6-2649872 and KASP-6-2919895, were identified placing the gene(s) in a 270 kb interval on chromosome 6 (**Table 3**; **Figure 1**).

**Table 3:** Segregation of locus among F_2_ lines that were critical to the mapping of *RRKN1*.

*RRKN1* interval on chromosome 6							
F_2_ lines*	6-1913541	6-2.232.949	6-2.649.872	*RRKN1*	6-2.919.895	6-3.298.501	6-3.340.952
11	SS	SS	SS	SS	SS	SS	SS
13	SS	SS	SS	SS	SS	**RS**	**RS**
16	RS	RS	RS	R	RS	RS	RS
17	RS	RS	RS	R	RS	RS	RS
18	SS	SS	SS	SS	SS	SS	SS
29	SS	SS	SS	SS	SS	SS	SS
35	**RS**	SS	SS	SS	SS	SS	SS
50	SS	SS	SS	SS	SS	SS	SS
59	SS	SS	SS	SS	SS	SS	SS
65	**RS**	**RS**	**RS**	SS	SS	SS	SS
66	**RS**	**RS**	**RS**	SS	**RS**	**RS**	**RS**
70	**RS**	SS	SS	SS	SS	SS	SS
71	SS	SS	SS	SS	SS	SS	SS
74	SS	SS	SS	SS	SS	SS	SS
75	SS	SS	SS	SS	SS	SS	SS
78	SS	SS	SS	SS	SS	SS	SS
105	**RS**	SS	SS	SS	SS	SS	SS
107	**RR**	**RS**	**RS**	SS	**RS**	**RS**	**RS**
109	SS	SS	SS	SS	SS	SS	SS
110	SS	SS	SS	SS	SS	SS	SS
113	**RS**	**RS**	**RS**	SS	**RS**	**RS**	**RS**
117	**RS**	**RS**	**RS**	SS	**RS**	**RS**	**RS**
121	SS	SS	SS	SS	SS	SS	SS
124	SS	SS	SS	SS	SS	SS	SS
130	SS	SS	SS	SS	SS	SS	SS

F_2_ lines were generated from the cross between the resistant and the susceptible cultivars. SS homozygous for susceptible parent allele; RR homozygous for resistant parent allele; RS heterozygous. Important recombinants are given in bold.

### The *RRKN1* interval contains defense-related genes

We identified genes within the interval for *RRKN1*, using the ITAG annotation of tomato reference genomes SL.3 and SL.4 (Hosmani et al, 2019). There are 28 predicted genes within the *RRKN1* interval according to ITAG 3.2 (**Table 4**) and 24 predicted genes according to ITAG version 4.0. The numbers of genes differ as genes *Solyc06g008770*.*2* (*Mi-1E*, *CNL4*), *Solyc06g008790.3* (*Mi-1F*, *Mi-1.1*) and *Solyc06g008800.2* (*Mi-1G, CNL6*) in ITAG 3.2 disappeared in ITAG 4.0 and two genes encoding transport inhibitor response 1 protein are also missing from ITAG 3.2.

**Table 4:** Genes within *RRKN1* interval according to ITAG 3.0.

Gene ID	Putative Function
Solyc06g008740.3	Zinc finger transcription factor 40
Solyc06g008750.1	Glutaredoxin
Solyc06g008760.1	Glutaredoxin
Solyc06g008770.2	Mi-E (CNL4)
Solyc06g008780.2	Transport inhibitor response 1
Solyc06g008790.3	Mi-F (Mi-1.1)
Solyc06g008800.2	Mi-G (CNL6)
Solyc06g008803.1	Transport inhibitor response 1-like protein
Solyc06g008805.1	Transport inhibitor response 1
Solyc06g008810.3	Transport inhibitor response 1
Solyc06g008807.1	Transport inhibitor response 1
Solyc06g008820.3	Na+/H+ antiporter 1
Solyc06g008830.1	Protein kinase superfamily protein
Solyc06g008840.3	BRCT domain-containing DNA repair protein, putative isoform 1
Solyc06g008850.3	DCD (Development and Cell Death) domain protein
Solyc06g008860.3	u1 small nuclear ribonucleoprotein C
Solyc06g008870.2	Gid1-like gibberellin receptor
Solyc06g008880.3	WD40 repeat-containing protein
Solyc06g008890.4	Kinase family protein
Solyc06g008900.3	RING/U-box superfamily protein
Solyc06g008910.2	Hexosyltransferase
Solyc06g008920.3	AMP-dependent synthetase/ligase
Solyc06g008930.3	MLP-like protein 31
Solyc06g008940.3	Elongation factor Tu
Solyc06g008950.2	Heavy metal transport/detoxification superfamily protein
Solyc06g008960.3	Mediator of RNA polymerase II transcription subunit 33A
Solyc06g008970.3	RNA helicase DEAH-box18
Solyc06g008980.3	F-box/WD repeat-containing 10

**Figure 2:**
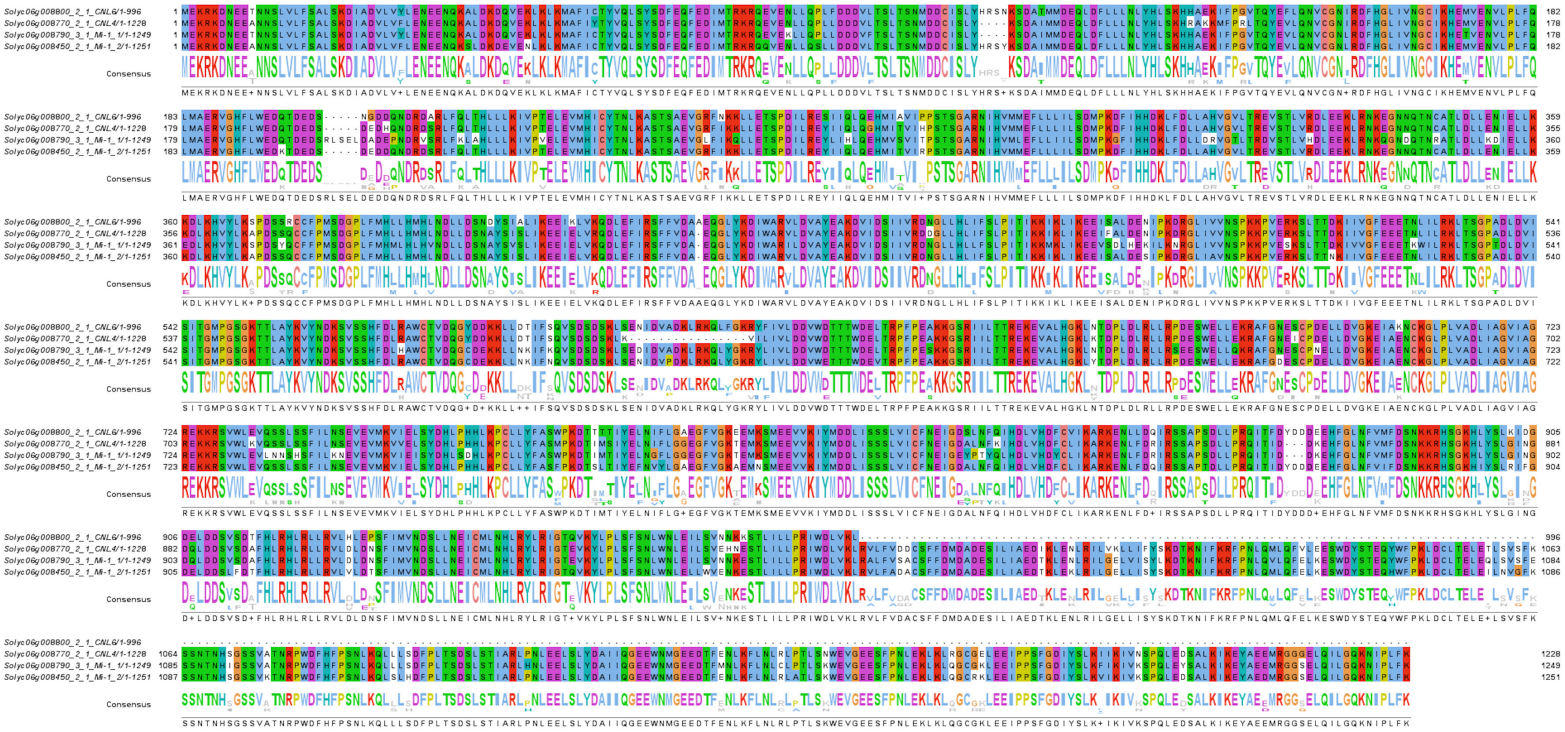
Multiple sequence alignment of Mi-1.2 and the three NLR proteins encoded in the 270-kb interval. Amino acid sequences were obtained from the International Tomato Genome Sequencing Project (https://solgenomics.net/organism/Solanum_lycopersicum/genome/) and aligned using Clustal Omega (Sievers et al., 2011) at the website of the European Bioinformatics Institute (McWilliam et al., 2013). The alignment image was rendered using Jalview (Waterhouse et al., 2009). The interval contains three possible candidates for *RRKN1*: *Mi-E*, *Mi-F* and *Mi-G.* In the SolGen genome annotation, these genes correspond to loci Solyc06g008770.2.1, Solyc06g008790.3.1 and Solyc06g008800.2.1 respectively. The respective GenBank protein accessions are ABI96216.1, ABI96217.1 and ABI96218.1.

The genes *Mi-1E, Mi-1F and Mi-1G* within the interval fall within a clade of nematode-specific *R* genes, as sequence similarity searches using BLASTP against the NCBI’s Non-Redundant Proteins database revealed highest degree of similarity with *R*-genes previously implicated in resistance to nematodes in tomato and pepper. As tomato accession MT12 does not carry a functional allele of *Mi-1.2*, the most likely candidates for *RRKN1* are *Mi-1E*, *Mi-F* and *Mi-1G*. Amino acid sequence alignments of these three putative *R*-proteins along with Mi-1.2 indicated these proteins are highly similar to each other but not identical (**Figure 2**).

### *RRKN1* gene region is heterozygous in parents

As the mapping population was generated by crossing two F_1_ parents, the genomic vicinity of the resistance gene is expected to be heterozygous. Therefore, we developed five new KASP markers were developed within the interval (**Supplementary Table 2**) and the markers were then used these to screen parents and the mapping population. The results confirmed the region to be heterozygous (**Supplementary Table 4**).

## Discussion

The *Mi-1.2* gene is widely used for resistance to root-knot nematodes; however, it has some significant drawbacks, including breakdown of resistance at high soil temperatures. Therefore, new resistance sources must be identified and incorporated into tomato breeding programmes. In this study, we demonstrate that resistance source MT12 does not carry a functional alle of *Mi-1.2* but provides durable resistance due to *RRKN1* at high soil temperatures. Additionally, MT12 does not confer resistance to naturally identified *Mi-1.2*-virulent isolates, indicating that *RRKN1* shares a similar resistance-spectrum to *Mi-1.2* but does not break down at high temperature. Also, as expected, the Seval F_1_ plant carrying the *Mi-1.2* gene was observed to lose resistance at high soil temperatures. In previous studies, different resistance genes such as *Mi-2*, *Mi-3*, *Mi-4*, *Mi-5*, *Mi-6* and *Mi-9* were determined against RKNs at high soil temperatures (Cap et al., 1993; Yaghoobi et al., 1995; Veremis and Roberts, 1996a; Veremis and Roberts, 1996b; Veremis et al., 1999; Ammiraju et al., 2003).

Ammiraju et al. (2003) found the LA2157 (*Mi-9*) accession to be resistant to avirulent populations of *M. arenaria*, *M. incognita* and *M. javanica* at 25°C and 32°C. Also, resistance was provided by a single dominant gene located in the vicinity of the *Mi-1.2* gene on the short arm of chromone 6. In another study, Jablonska et al. (2007) suggested that *Mi-9*, which is a heat-stable resistance gene, was located on chromosome 6 and may be a homologue of *Mi*-*1*. Recently, Wang et al. (2013) reported that *Mi-HT*, conferring resistance to *M. incognita* at high temperatures (32°C) in line ZN17, was a single dominant gene and located on the short arm of chromosome 6. Also, molecular data indicated that *Mi-HT* might be an orthologue of *Mi-1* and *Mi-9* or a new gene. Gene *Mi-9* is located on chromosome 6 between positions 2305101 and 3327863 and *Mi-HT* is between 2305101 bp and 2327783 bp, based on investigating positions of published markers linked to *Mi-9* and *Mi-HT* genes in version SL3.1 genome assembly of tomato. In the present study, we showed that the *RRKN1* gene is on chromosome 6 and in a 270-kb interval between positions 2.649.872 and 2.919.895. Recently, Jiang et al. (2023) determined that the *Mi-9* gene is located between nucleotides 3.113.154 and 3.116.774 on chromosome 6 of LA2157. They also showed that the similarity of *Mi-1.2* and *Mi-9* proteins was 97.22%.

Previously, we mapped *Pvr4* in pepper (Devran et al., 2015), *Frl* in tomato (Devran et al., 2018) and *CsCvy-1* in cucumber (Kahveci et al., 2021) using genomics coupled with the bulked segregant analysis method. In this study, to determine the location of *RRKN1*, we developed molecular markers to cover all chromosomes and used them to screen F_2_ populations. Narrowing the genomic interval depends on the number of recombinants in a given mapping population. In the mapping exercise of *Frl* gene in tomato, we used 542 F_2_ lines and mapped the gene to a 900-kb interval on chromosome 9 (Devran et al., 2018). In this study, we used only 130 F_2_ lines and determined a 270-kb interval for *RRKN1.* This improved genomic resolution may have been due to the fact that F_1_ lines in a commercial programme are produced from pure breeding lines, which would have undergone many recombination events due to continuous crossing to accumulate the desired traits. This in turn, may have helped identification of the desired recombinant lines for mapping. As we had a few more recombinants from either side of the flanking markers, we attempted to narrow the interval further and developed markers tightly linked to the gene. Results of implementing newly developed KASP markers proved difficult due to the heterozygous nature of the *RRKN1* interval. In some cases, the number of the recombination in a region could be influenced by chromosome rearrangement and recombination suppression (Tang et al., 2008; Verlaan et al., 2011).

The interval contains three possible candidates for *RRKN1*: *Mi-E*, *Mi-F* and *Mi-G.* In the SolGen genome annotation, these genes correspond to loci Solyc06g008770.2.1, Solyc06g008790.3.1 and Solyc06g008800.2.1 respectively. The amino acid alignment of these putative disease resistance proteins show that they are very similar to each other as well as to the well-known Mi-1.2. Recent studies show that the NLR proteins work as a pair. For example, *Arabidopsis* RPP2 family (RPP2A, RPP2B, RPP2C and RPP2D) have been shown to be required for the recognition of ATR2 effector for the downy mildew pathogen (Kim et al., 2023). Similarly, *Arabidopsis* TIR-NLR RRS1 and RPS4 (Guo et al., 2020), and the rice CC-NLR pairs RGA4/RGA5 and Pik-1/Pik-2 (Zdrzałek et al., 2020) have been reported to work together. As we did not proceed to clone the functional gene for *RRKN1*, further studies would be required to reveal which one(s) of these three candidate genes are providing the resistance against the RKNs. Possible approaches that can be utilized may include CRISPR/Cas9 system (Erdoğan et al., 2023), virus-induced gene silencing (VIGS) method (Valentine et al., 2004), cloning each of them and transforming to a susceptible line and assaying the lines with a relevant nematode population. Once the gene is identified, a new MAS-friendly marker could be developed withing the gene to use in the breeding programme.

In summary, root-knot nematodes are an important problem for tomato growing areas. The use of resistant cultivars is one of the most attractive disease management methods. The *Mi-1.2* gene has been used for a long time in tomatoes. However, *Mi-1.2* gene was overcome by *Mi-1.2*-virulent RKN populations (Roberts, 1990; Castagnone-Sereno et al., 1994; Kaloshian et al., 1996; Devran and Söğüt, 2010) and loses its effect at high soil temperatures (Dropkin, 1969; Özalp and Devran, 2018). Molecular marker-assisted selection of a resistant gene is important for breeding programmes. In conclusion, identifying a new gene such as *RRKN1* and developing molecular markers tightly linked to gene, discovered in the present study, can help generate new tomato varieties, fine-mapping and cloning of the gene. Growers can use *RRKN1*-carrying lines instead of tomato varieties bearing the *Mi-1.2* resistance that breaks down at high soil temperature. In addition, pyramiding of *RRKN1* and *Mi-1.2* genes in superior tomato lines would prolong controlling of the root-knot nematodes.

## Data availability statement

The datasets presented in this study can be found in online repositories. The names of the repository/repositories and accession number(s) can be found in the article/**Supplementary Material**.

## Author contributions

ZD: Conceptualization, Data curation, Formal Analysis, Funding acquisition, Methodology, Project administration, Supervision, Validation, Writing – original draft, Writing – review & editing. TÖ: Data curation, Formal Analysis, Methodology, Writing – review & editing. DS: Formal Analysis, Writing – review & editing. MT: Conceptualization, Formal Analysis, Funding acquisition, Supervision, Writing – original draft, Writing – review & editing.

## References

[B1] Abd-Elgawad M. M. M. (2022). Understanding molecular plant–nematode interactions to develop alternative approaches for nematode control. Plants 11, 2141. doi: 10.3390/plants11162141 36015444 PMC9415668

[B2] Ammiraju J. Veremis J. Huang X. Roberts P. Kaloshian I. (2003). The heat-stable root-knot nematode resistance gene *Mi-9* from *Lycopersicon Peruvianum* is localized on the short arm of chromosome 6. Theor. Appl. Genet. 106, 478–484. doi: 10.1007/s00122-002-1106-y 12589548

[B3] Barragan A. C. Weigel D. (2021). Plant NLR diversity: the known unknowns of pan-NLRomes. Plant Cell 33, 814–831. doi: 10.1093/plcell/koaa002 33793812 PMC8226294

[B4] Cap G. B. Roberts P. Thomason I. (1993). Inheritance of heat-stable resistance to *Meloidogyne incognita* in *Lycopersicon Peruvianum* and its relationship to the Mi gene. Theor. Appl. Genet. 85, 777–783. doi: 10.1007/BF00225019 24196050

[B5] Castagnone-Sereno P. Bongiovanni M. Dalmasso A. (1994). Reproduction of virulent isolates of *Meloidogyne incognita* on susceptible and *Mi*-resistant tomato. J. Nematol. 26, 324–328. 19279899 PMC2619507

[B6] Devran Z. Kahveci E. (2019). Development and validation of a user-friendly KASP marker for the *Sw-5* locus in tomato. Australas. Plant Pathol. 48, 503–507. doi: 10.1007/s13313-019-00651-1

[B7] Devran Z. Kahveci E. Hong Y. Studholme D. J. Tör M. (2018). Identifying molecular markers suitable for *Frl* selection in tomato breeding. Theor. Appl. Genet. 131, 2099–2105. doi: 10.1007/s00122-018-3136-0 29982848 PMC6154021

[B8] Devran Z. Kahveci E. Özkaynak E. Studholme D. J. Tör M. (2015). Development of molecular markers tightly linked to *Pvr4* gene in pepper using next-generation sequencing. Mol. Breed. 35, 1–9. doi: 10.1007/s11032-015-0294-5 PMC4361654 25798050

[B9] Devran Z. Sogut M. A. (2009). Distribution and identification of root-knot nematodes from Turkey. J. Nematol. 41, 128–133. 22661785 PMC3365313

[B10] Devran Z. Söğüt M. A. (2010). Occurrence of virulent root-knot nematode populations on tomatoes bearing the *Mi* gene in protected vegetable-growing areas of Turkey. Phytoparasitica 38, 245–251. doi: 10.1007/s12600-010-0103-y

[B11] Dropkin V. (1969). The necrotic reaction of tomatoes and other hosts resistant to *Meloidogyne*: reversal by temperature. Phytopathology 59, 1632–1637.

[B12] Du C. Jiang J. Zhang H. Zhao T. Yang H. Zhang D.. (2020). Transcriptomic profiling of *Solanum Peruvianum* LA3858 revealed a Mi-3-mediated hypersensitive response to *Meloidogyne incognita* . BMC Genomics 21, 250. doi: 10.1186/s12864-020-6654-5 32293256 PMC7092525

[B13] Erdoğan İ. Cevher-Keskin B. Bilir Ö. Hong Y. Tör M. (2023). Recent developments in CRISPR/Cas9 genome-editing technology related to plant disease resistance and abiotic stress tolerance. Biology 12, 1037. doi: 10.3390/biology12071037 37508466 PMC10376527

[B14] Fernandez-Pozo N. Menda N. Edwards J. D. Saha S. Tecle I. Y. Strickler S. R.. (2015). The Sol Genomics Network (SGN) from genotype to phenotype to breeding. Nucleic Acids Res. 43, 1036–1041. doi: 10.1093/nar/gku1195 PMC4383978 25428362

[B15] Guo H. Ahn H. Y. Sklenar J. Huang J. Ma Y. Ding P.. (2020). Phosphorylation-regulated activation of the Arabidopsis RRS1-R/RPS4 immune receptor complex reveals two distinct effector recognition mechanisms. Cell Host Microbe 27, 769–781.e6. doi: 10.1016/j.chom.2020.03.008 32234500

[B16] Gururani M. A. Venkatesh J. Upadhyaya C. P. Nookaraju A. Pandey S. K. Park S. W. (2012). Plant disease resistance genes: current status and future directions. Physiol. Mol. Plant Pathol. 78, 51–65. doi: 10.1016/j.pmpp.2012.01.002

[B17] Hashem M. Abo-Elyousr K. A. (2011). Management of the root-knot nematode *Meloidogyne incognita* on tomato with combinations of different biocontrol organisms. Crop Protect. 30, 285–292. doi: 10.1016/j.cropro.2010.12.009

[B18] Hosmani P. S. Flores-Gonzalez M. van de Geest H. Maumus M. Bakker L. V. Schijlen E.. (2019). An improved de novo assembly and annotation of the tomato reference genome using single-molecule sequencing, Hi-C proximity ligation and optical maps. bioRxiv, 767764. doi: 10.1101/767764

[B19] Jablonska B. Ammiraju J. S. Bhattarai K. K. Mantelin S. De Ilarduya O. M. Roberts P. A.. (2007). The *Mi-9* gene from *Solanum arcanum* conferring heat-stable resistance to root-knot nematodes is a homolog of *Mi-1* . Plant Physiol. 143, 1044–1054. doi: 10.1104/pp.106.089615 17172289 PMC1803715

[B20] Jiang L. Ling J. Zhao J. Yang Y. Yang Y. Li Y.. (2023). Chromosome-scale genome assembly-assisted identification of Mi-9 gene in Solanum arcanum accession LA2157, conferring heat-stable resistance to *Meloidogyne incognita* . Plant Biotechnol. J. 21, 1496–1509. doi: 10.1111/pbi.14055 37074757 PMC10281608

[B21] Kahveci E. Devran Z. Özkaynak E. Hong Y. Studholme D. J. Tör M. (2021). Genomic-assisted marker development suitable for *Cscvy-1* selection in cucumber breeding. Front. Plant Sci. 12. doi: 10.3389/fpls.2021.691576 PMC8416629 34489994

[B22] Kaloshian I. Williamson V. Miyao G. Lawn D. Westerdahl B. (1996). “Resistance-breaking” nematodes identified in California tomatoes. Calif. Agric. 50, 18–19. doi: 10.3733/ca.v050n06p18

[B23] Kaloshian I. Yaghoobi J. Liharska T. Hontelez J. Hanson D. Hogan P.. (1998). Genetic and physical localization of the root-knot nematode resistance locus *Mi* in tomato. Mol. Gen. Genet. 257, 376–385. doi: 10.1007/s004380050660 9520273

[B24] Kim D. S. Woods-Tör A. Cevik V. Furzer O. J. Li Y. Ma W.. (2023). ATR2^Cala2^ from *Arabidopsis*-infecting downy mildew requires 4 TIR-NLR immune receptors for full recognition. bioRxiv, 538220. doi: 10.1101/2023.04.25.538220 38742296

[B25] Li H. Handsaker B. Wysoker A. Fennell T. Ruan J. Homer N.. (2009). The sequence alignment/map format and SAMtools. Bioinformatics 25, 2078–2079. doi: 10.1093/bioinformatics/btp352 19505943 PMC2723002

[B26] Marquez J. Hajihassani A. (2023). Successional effects of cover cropping anddeep tillage on suppression of plant-parasitic nematodes and soilborne fungal pathogen. Pest Manage. Sci. 79, 2737–2747. doi: 10.1002/ps.7450 36914802

[B27] Mchale L. Tan X. Koehl P. Michelmore R. W. (2006). Plant NBS-LRR proteins: adaptable guards. Genome Biol. 7, 212. doi: 10.1186/gb-2006-7-4-212 16677430 PMC1557992

[B28] McWilliam H. Li W. Uludag M. Squizzato S. Park Y. M. Buso N.. (2013). Analysis tool web services from the EMBL-EBI. Nucleic Acids Res. 41 (Web Server issue), W597–W600. doi: 10.1093/nar/gkt376 23671338 PMC3692137

[B29] Milligan S. B. Bodeau J. Yaghoobi J. Kaloshian I. Zabel P. Williamson V. M. (1998). The root knot nematode resistance gene *Mi* from tomato is a member of the leucine zipper, nucleotide binding, leucine-rich repeat family of plant genes. Plant Cell 10, 1307–1319. doi: 10.1105/tpc.10.8.1307 9707531 PMC144378

[B30] Nas Y. Özalp T. Devran Z. (2023). Screening of Urfa pepper landraces for resistance to *Meloidogyne incognita* . J. Plant Dis. Prot. 130, 77–83. doi: 10.1007/s41348-022-00673-w

[B31] Öçal S. Özalp T. Devran Z. (2018). Reaction of wild eggplant *Solanum torvum* to different species of root-knot nematodes from Turkey. J. Plant Dis. Prot. 125, 577–580. doi: 10.1007/s41348-018-0167-3

[B32] Özalp T. Devran Z. (2018). Response of tomato plants carrying *Mi-1* gene to *Meloidogyne incognita* (Kofoid & White 1919) chitwood 1949 under high soil temperatures. Turkish J. Entomol. 42, 313–322. doi: 10.16970/entoted.467189

[B33] Phani V. Khan M. R. Dutta T. K. (2021). Plant-parasitic nematodes as a potential threat to protected agriculture: Current status and management options. Crop Prot. 144, 105573. doi: 10.1016/j.cropro.2021.105573

[B34] Rawal S. (2020). A review on root-knot nematode infestation and its management practices through different approaches in tomato. Trop. Agroecosystem 1, 92–96. doi: 10.26480/taec.02.2020.92.96

[B35] Roberts P. (1990). “Resistance to nematodes: definitions, concepts, and consequences,” in Methods for evaluating plant species for resistance to plant-parasitic nematodes. Ed. Star J. L. (Maryland, USA: Society of Nematologists, Hyattsville), 1–15.

[B36] Roberts P. Thomason J. (1986). Variability in reproduction of isolates of *Meloidogyne incognita* and *M. javanica* on resistant tomato genotypes. Plant Dis. 70, 547–551. doi: 10.1094/PD-70-547

[B37] Robinson J. T. Thorvaldsdóttir H. Winckler W. Guttman M. Lander E. S. Getz G.. (2011). Integrative genomics viewer. Nat. Biotechnol. 29, 24–26. doi: 10.1038/nbt.1754 21221095 PMC3346182

[B38] Seah S. Williamson V. M. Garcia B. E. Mejia L. Salus M. S. Martin C. T.. (2007). Evaluation of a codominant SCAR marker for detection of the *Mi-1* locus for resistance to root-knot nematode in tomato germplasm. Tomato Genet. Cooperative Rep. 57, 37–40.

[B39] Sievers F. Wilm A. Dineen D. G. Gibson T. J. Karplus K. Li W.. (2011). Fast, scalable generation of high-quality protein multiple sequence alignments using Clustal Omega. Mol. Syst. Biol. 7, 539. doi: 10.1038/msb.2011.75 21988835 PMC3261699

[B40] Talavera M. Verdejo-Lucas S. Ornat C. Torres J. Sorribas F. (2009). Crop rotations with *Mi* gene resistant and susceptible tomato cultivars for management of root-knot nematodes in plastic houses. Crop Prot. 28, 662–667. doi: 10.1016/j.cropro.2009.03.015

[B41] Tang X. Szinay D. Lang C. Ramanna M. S. van der Vossen E. A. Datema E.. (2008). Cross-species bacterial artificial chromosome-fluorescence in *situ* hybridization painting of the tomato and potato chromosome 6 reveals undescribed chromosomal rearrangements. Genetics 180, 1319–1328. doi: 10.1534/genetics.108.093211 18791231 PMC2581937

[B42] Valentine T. Shaw J. Blok V. C. Phillips M. S. Oparka K. J. Lacomme C. (2004). Efficient virus-induced gene silencing in roots using a modified tobacco rattle virus vector. Plant Physiol. 136, 3999–4009. doi: 10.1104/pp.104.051466 15591447 PMC535832

[B43] Veremis J. Roberts P. (1996a). Identification of resistance to *Meloidogyne javanica* in the *Lycopersicon Peruvianum* complex. Theor. Appl. Genet. 93, 894–901. doi: 10.1007/BF00224091 24162423

[B44] Veremis J. Roberts P. (1996b). Relationships between *Meloidogyne incognita* resistance genes in *Lycopersicon Peruvianum* differentiated by heat sensitivity and nematode virulence. Theor. Appl. Genet. 93, 950–959. doi: 10.1007/BF00224098 24162430

[B45] Veremis J. Roberts P. (2000). Diversity of heat-stable genotype specific resistance to *Meloidogyne* in Maranon races of *Lycopersicon Peruvianum* complex. Euphytica 111, 9–16. doi: 10.1023/A:1003776201585

[B46] Veremis J. Van Heusden A. Roberts P. (1999). Mapping a novel heat-stable resistance to *Meloidogyne* in *Lycopersicon Peruvianum* . Theor. Appl. Genet. 98, 274–280. doi: 10.1007/s001220051068

[B47] Verlaan M. G. Szinay D. Hutton S. F. de Jong H. Kormelink R. Visser R. G.. (2011). Chromosomal rearrangements between tomato and *Solanum Chilense* hamper mapping and breeding of the TYLCV resistance gene Ty-1. Plant J. 68, 1093–1103. doi: 10.1111/j.1365-313X.2011.04762.x 21883550

[B48] Wang Y. Yang W. Zhang W. Han Q. Feng M. Shen H. (2013). Mapping of a heat-stable gene for resistance to southern root-knot nematode in Solanum lycopersicum. Plant Mol. Biol. Rep. 31, 352–362. doi: 10.1007/s11105-012-0505-8

[B49] Waterhouse A. M. Procter J. B. Martin D. M. A. Clamp M. Barton G. J. (2009). Jalview Version 2 - A multiple sequence alignment editor and analysis workbench. Bioinformatics 25, 1189–1191. doi: 10.1093/bioinformatics/btp033 19151095 PMC2672624

[B50] Williamson V. M. Hussey R. S. (1996). Nematode pathogenesis and resistance in plants. Plant Cell 8, 1735–1745. doi: 10.1105/tpc.8.10.1735 8914324 PMC161311

[B51] Wu W. W. Shen H. L. Yang W. C. (2009). Sources for heat-stable resistance to southern root-knot nematode (*Meloidogyne incognita*) in *Solanum lycopersicum* . Agric. Sci. China 8, 697–702. doi: 10.1016/S1671-2927(08)60267-9

[B52] Yaghoobi J. Kaloshian I. Wen Y. Williamson V. (1995). Mapping a new nematode resistance locus in *Lycopersicon Peruvianum* . Theor. Appl. Genet. 91, 457–464. doi: 10.1007/BF00222973 24169835

[B53] Zdrzałek R. Kamoun S. Terauchi R. Saitoh H. Banfield M. J. (2020). The rice NLR pair Pikp-1/Pikp-2 initiates cell death through receptor cooperation rather than negative regulation. PloS One 15, e0238616. doi: 10.1371/journal.pone.0238616 32931489 PMC7491719

