# Supplementary material for: Mapping of the gene in tomato conferring resistance to root-knot nematodes at high soil temperature
Source: Front Plant Sci. 2023 Oct 10;14:1267399. doi: 10.3389/fpls.2023.1267399 (PMC10602802; doi:10.3389/fpls.2023.1267399)
Supplement: Supplementary file 1 [file Image_1.pdf]

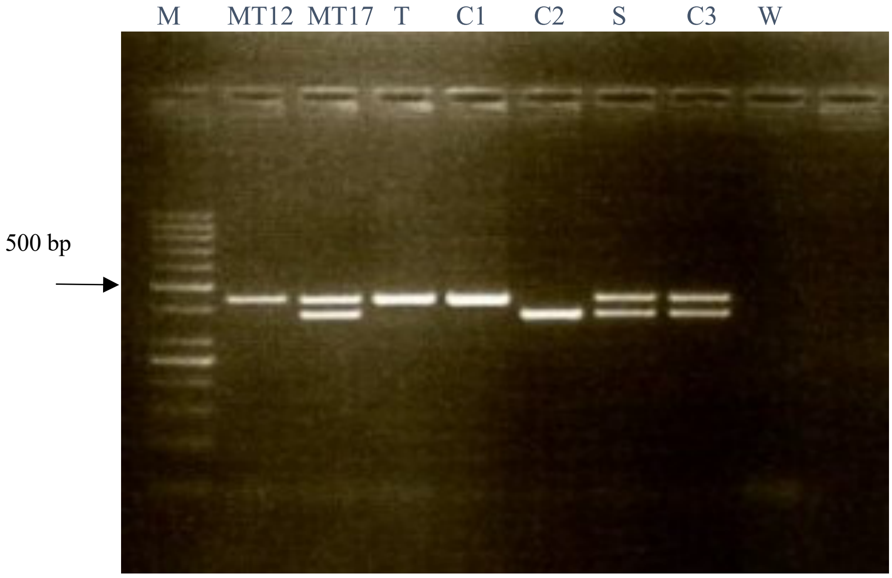

Supplementary Figure 1. PCR products of tomato plants using the Mi23 primer set that amplify *Mi-1.2.* M: Molecular marker (GRISP DNA Ladder 50 bp), MT12: Resistant parent, MT17: Susceptible parent, T:Tueza F_1 (Susceptible cultivar, SS), C1: Susceptible control (SS), C2: Homozygous resistant (RR), S:Seval F_1 (Heterozygous resistant cultivar, RS) C3: Heterozygous control (RS), W: Water. For Mi23 pirmer set; RR: 380 bp, SS:430 bp, RS: 430 bp and 380 bp
